# Research Progress in Applications of the CRISPR-Cas12b System in Pathogen Nucleic Acid Detection

**DOI:** 10.3390/cimb48090875

**Published:** 2026-08-28

**Authors:** Jiangying Li, Xueyong Zhang

**Affiliations:** 1 The Academy of Animal and Veterinary Sciences, Qinghai University, Xining 810016, China; ljiangying@126.com; 2 Qinghai Provincial Key Laboratory of Pathogen Diagnosis for Animal Disease and Green Technical Research for Prevention and Control, Xining 810016, China

**Keywords:** CRISPR-Cas12b, pathogen nucleic acid detection, molecular diagnosis, applied research

## Abstract

This review systematically summarizes recent advances in CRISPR-Cas12b-based pathogen nucleic acid detection. Starting with an overview of conventional nucleic acid detection methods and core CRISPR-Cas mechanisms, we highlight the unique properties that distinguish Cas12b from other Cas effectors. We elaborate on the working principles of the CRISPR-Cas12b system and present comparative structural and functional analyses with other Cas variants to underscore its distinctive molecular architecture and enzymatic properties. A comprehensive evaluation of current applications demonstrates the efficacy of CRISPR-Cas12b-based diagnostics across diverse pathogens, including viruses, bacteria, and parasites, with a specific focus on its integration with isothermal amplification techniques. We further examine the translational potential of CRISPR-based diagnostics in clinical settings, while critically analyzing persistent technical challenges including off-target effects, signal amplification limitations, and sample preparation requirements. Targeted strategic recommendations are proposed to optimize detection sensitivity, develop multiplexed detection platforms, and implement point-of-care testing configurations. This review aims to systematically correlate the unique characteristics of Cas12b with its broad diagnostic applications, thereby addressing key research gaps in its progression toward clinical validation and field deployment. It also provides a targeted framework to accelerate the translation of this technology from laboratory platforms to practical diagnostic solutions.

## 1. Introduction

As a pivotal technology for diagnosing pathogenic infections, nucleic acid detection plays a critical role in medicine and biology. Traditional nucleic acid detection methods, particularly polymerase chain reaction (PCR) and quantitative real-time PCR (qPCR), have become the gold standard for pathogen detection and are widely utilized in clinical practice and scientific research due to their high sensitivity and reliability. Moreover, these techniques are indispensable in the development of emerging detection systems. For instance, qPCR is a core tool for verifying the efficiency of CRISPR-based detection methods. Nevertheless, their application in point-of-care testing scenarios remains limited by the reliance on specialized equipment and relatively long processing times [1]. In recent years, the CRISPR (Clustered Regularly Interspaced Short Palindromic Repeats) system has emerged as a transformative nucleic acid detection platform [1]. CRISPR systems were originally identified as prokaryotic adaptive immune systems that recognize and cleave specific DNA sequences, and were divided into Class 1 and Class 2 based on their effector protein architectures [2]. Among Class 2 systems, Cas12b (C2c1) has garnered significant attention due to its compact size, exceptional specificity, and robust thermal stability, demonstrating considerable potential for nucleic acid diagnostics [2,3].

The CRISPR-Cas12b system integrates isothermal amplification with the sequence-specific recognition capabilities of the Cas12b nuclease, enabling rapid nucleic acid amplification and detection under constant-temperature conditions [3]. This innovative approach circumvents the thermocycling requirements of traditional PCR, offering distinct advantages in operational simplicity, detection speed, specificity, and sensitivity [4]. Mechanistically, target nucleic acids undergo isothermal amplification to generate abundant target sequences [5]. Guided by sequence-specific single-guide RNA (sgRNA), the Cas12b protein recognizes and cleaves these target sequences, while simultaneously activating collateral cleavage activity against surrounding non-target single-stranded DNA (ssDNA) reporters [6]. This dual cleavage activity generates measurable fluorescence signals or visual readouts, enabling rapid and sensitive detection of pathogens.

Current advancements in CRISPR-Cas12b technology have shown considerable promise in clinical diagnostics. The system has been effectively implemented for detecting diverse pathogens, including *Klebsiella pneumoniae*, *Toxoplasma gondii*, *Chlamydia psittaci* and *Mycoplasma pneumoniae*, demonstrating broad applicability and diagnostic accuracy [7]. Currently, optimization research on the CRISPR-Cas12b system mainly focuses on enhancing amplification efficiency, optimizing sgRNA design, and developing signal-amplification strategies, aiming to break through the bottlenecks of its sensitivity and specificity. Meanwhile, detection methods based on this system are being extended to fields such as clinical diagnosis, food safety, and environmental pathogen monitoring, providing key technical support for disease diagnosis, treatment, and prevention [8,9]. In this review, we first elucidate the molecular architecture, core catalytic mechanism, and unique performance strengths of Cas12b, alongside a comparative analysis of its structural and functional properties relative to other representative Cas effectors. We then systematically summarize the latest research progress in Cas12b-mediated detection of viral, bacterial, and parasitic pathogens. Finally, we provide an in-depth discussion of prevailing technical bottlenecks and outline key future directions for clinical translation, offering a clear developmental roadmap and conceptual framework to guide sustained innovation in this field.

## 2. CRISPR-Cas12b

### 2.1. Basics of CRISPR Technology

**Figure 1:**
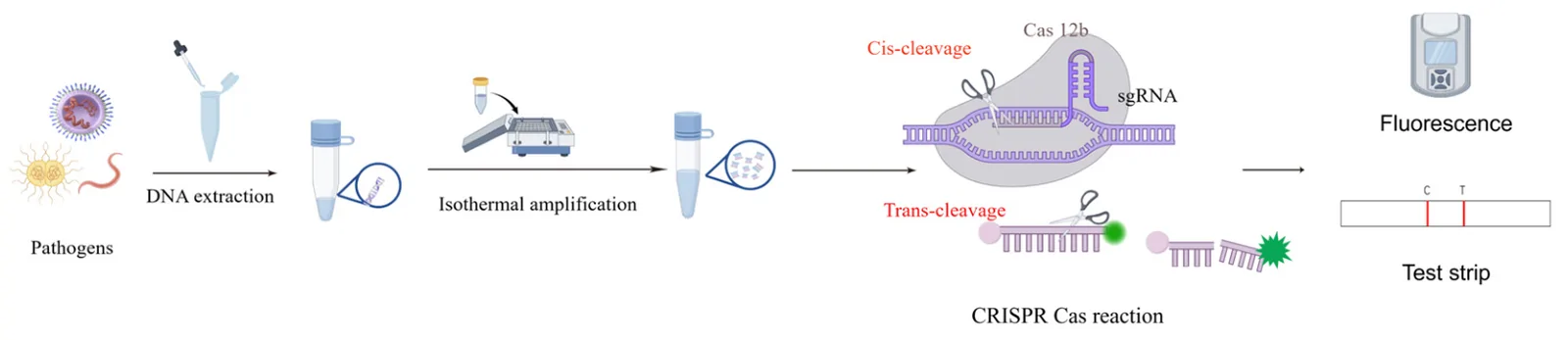
Flowchart of CRISPR-Cas12b-based Pathogenic Nucleic Acid Detection. (Created By Figdraw (https://www.figdraw.com/#/)): CRISPR-based detection can target the nucleic acids of various pathogens, including bacteria, viruses, parasites, and other targets.

CRISPR technology is a revolutionary genome-editing tool derived from the adaptive immune system of prokaryotes, primarily bacteria and archaea [10]. This system enables these organisms to recognize and defend against foreign genetic material, such as viruses, by storing segments of their DNA as a record of past infections. The CRISPR system consists of two key components: CRISPR-associated (Cas) proteins and CRISPR RNA (crRNA) [11]. While CRISPR-Cas naturally functions as a bacterial adaptive immune system, this core molecular machinery has been repurposed by researchers to achieve programmable genome editing in vitro and in eukaryotic cells. The crRNA guides the Cas protein to the target DNA sequence, where the Cas protein, typically a nuclease, introduces double-strand breaks, leading to gene editing through repair mechanisms such as non-homologous end joining or homology-directed repair. The versatility and precision of CRISPR technology have made it a cornerstone in molecular biology, allowing for not only genome editing but also applications in diagnostics, particularly in the detection of nucleic acids [12]. The CRISPR-Cas12b variant, in particular, has gained attention due to its unique properties. Its molecular weight (110–130 kDa) facilitates cellular delivery, and its high specificity, coupled with flexible protospacer adjacent motif (PAM) recognition, enables single-nucleotide discrimination under optimized conditions. However, discrimination efficiency depends critically on guide RNA design parameters, including mismatch position, reaction temperature, and buffer composition. Additionally, its remarkable thermostability, maintaining activity across 37–65 °C, enables seamless integration with isothermal amplification. These features enhance its utility in various applications, including pathogen detection and gene editing [13,14]. Figure 1 shows the flowchart of CRISPR-Cas12b-based pathogenic nucleic acid detection. Compared with traditional nucleic acid detection methods, CRISPR-Cas12b features shorter detection duration, lower dependence on large instruments, higher sensitivity and superior specificity, and thus serves as an efficient new platform for rapid pathogen nucleic acid testing. Table 1 details the differences between traditional nucleic acid detection methods and the CRISPR-Cas12b detection method in various dimensions.

Despite the outstanding comprehensive performance of CRISPR-Cas12b summarized above, the existing literature shows notable methodological discrepancies. Most studies only verify its detection performance under ideal laboratory buffer systems, while few systematically evaluate its anti-interference ability against complex clinical matrices such as plasma, feces and tissue homogenates. In addition, the optimal spacer length and sgRNA scaffold design schemes proposed by different research groups are inconsistent, and there is a lack of unified standardized operation parameters, which restricts the repeatability of this technology in clinical translation.

### 2.2. Molecular Mechanisms of Cas12b Function

Cas12b relies on two core functional modules to execute all enzymatic reactions: the recognition (REC) lobe and the catalytic RuvC domain. The coordinated conformational rearrangement of these two modules governs its cleavage activity [15,16]. The REC lobe contains multiple conserved α-helix and β-fold motifs, which are responsible for binding sgRNA and mediating precise base pairing between the spacer region and target DNA; the RuvC domain acts as the sole catalytic center of Cas12b, simultaneously undertaking the functions of target cis-cleavage and non-specific collateral trans-cleavage [17,18].

The activation of Cas12b follows a strict conformational cascade process: sgRNA first binds stably to the REC lobe to form ribonucleoprotein (RNP) complexes, and the whole protein maintains an inactive closed conformation. When the RNP complex recognizes and combines double-stranded DNA with the correct TTN PAM sequence, the complementary chain of the target inserts into the gap between REC and RuvC domains, triggering large-scale structural rearrangement of the REC lobe. This conformational change opens the substrate binding pocket of the RuvC domain and initiates targeted cis-cutting of double-stranded DNA. After completing the cleavage of the target template, the RuvC domain maintains an open active state and loses sequence recognition selectivity, enabling non-specific degradation of nearby single-stranded DNA substrates. This property constitutes the core molecular mechanism underlying signal generation in Cas12b-based diagnostic platforms [19,20].

The wide temperature tolerance (37–65 °C) of Cas12b originates from its unique tertiary protein structure: the interior forms a compact hydrophobic core to slow down thermal unfolding; abundant intramolecular salt bridges between charged amino acid residues stabilize the overall folding framework under high temperature; meanwhile, short rigid loops replace disordered flexible segments in key functional regions to suppress heat-induced protein denaturation and activity attenuation [21,22]. The above three structural characteristics together endow Cas12b with excellent heat resistance, and make it perfectly compatible with various isothermal amplification reactions.

**Table 1:** Comparison between Conventional Nucleic Acid Detection Methods and CRISPR-Cas12b Detection Method.

Evaluation Dimension	Conventional Nucleic Acid Detection Methods (Represented by PCR/qPCR)	CRISPR-Cas12b Detection Method
Detection Time	90~180 min	20–80 min
Equipment Dependence	High (relies on PCR instruments, fluorescence quantitative detectors and other laboratory-specific equipment)	Low (compatible with test strips, portable fluorometers, and even naked-eye reading)
Detection Sensitivity	Conventional PCR: 10^3^~10^4^ copies/T; qPCR: 10^1^~10^2^ copies/T (limited by amplification efficiency)	Extremely high, reaching the aM level (suitable for samples with low pathogen load)
Detection Specificity	High (relies on primer specificity), but there is a risk of cross-reaction in closely related species or subtypes	High (single-base resolution, Cas12b trans-cleavage is only activated by specific targets)
Multiplex Detection Capability	Mature (qPCR can detect multiple targets simultaneously)	To be optimized (currently limited by sgRNA orthogonality, there is a risk of signal crosstalk in multiplex detection)
Operation Complexity	Relatively high (requires professional personnel, step-by-step operation is prone to contamination)	Low (supports “one-tube one-step method”, easy for on-site personnel to master)
Cost	High initial investment in instruments, relatively low cost for a single qPCR reaction	The current reagent cost is relatively high, but there is no need for expensive instruments, and the cost reduction potential is great after large-scale production
Applicability	Suitable for laboratories, difficult to meet the on-site rapid detection needs of sudden public health events	Suitable for both laboratories and fields, lyophilized reagents support deployment in resource-limited areas
References	[23,24,25,26]	[27,28,29]

### 2.3. Characteristics and Advantages of Cas12b

Distinct from Cas9, Cas12a, and other Cas subtypes, Cas12b possesses multiple unique performance advantages. Its prominent thermostability enables compatibility with loop-mediated isothermal amplification (LAMP) and other isothermal amplification technologies, and the developed rapid detection kits can be deployed in primary healthcare and resource-limited areas without precise temperature control equipment [28]. However, current research on the “thermal stability” of Cas12b mostly focuses on verifying its initial activity at high temperatures, lacking systematic quantitative data on the rate of activity decay under long-term high-temperature reaction conditions. This activity decay may lead to a decrease in signal intensity during actual “one-pot” combinations, constituting a hidden bottleneck for technology integration [30]. In addition, Cas12b orthologs exhibit diverse PAM requirements, which allows flexible selection of genomic target sites. Combined with potent trans-cleavage activity, Cas12b can achieve high-sensitivity and high-specificity detection of pathogens and gene mutations [31,32]. More importantly, the strict base matching requirement of crRNA enables Cas12b to distinguish single-nucleotide polymorphisms accurately. Even a single-nucleotide difference can be identified through this precise pairing. For example, in the detection of viral variants (such as different variants of the novel coronavirus), Cas12b can, by virtue of this property, accurately distinguish nucleic acid sequences of different variants that only have single-nucleotide differences [27,33]. After binding the target, Cas12b triggers non-specific cleavage of single-stranded reporter DNA, producing signal cascade amplification and greatly improving detection sensitivity [34].

Collateral trans-cleavage activity constitutes the core functional feature and technical foundation that underpins the application of Cas12b in nucleic acid diagnosis [15,16]. When the Cas12b-sgRNA/crRNA ribonucleoprotein (RNP) complex specifically recognizes and binds target double-stranded DNA (dsDNA), it not only executes cis-cleavage of the target template but also simultaneously activates its promiscuous single-stranded DNA (ssDNA) cleavage capacity [16]. This target-triggered non-specific nuclease behavior is termed “trans-cleavage” or “collateral cleavage”. In practical diagnostic assays, fluorescent-quenched ssDNA reporter molecules are incorporated into the reaction system. Once Cas12b is allosterically activated upon target engagement, it indiscriminately degrades these reporter probes, releasing free fluorophores and generating measurable fluorescent readouts that enable ultrasensitive detection of trace target molecules [15,35]. This “target-activated, non-specific signal amplification” mechanism lays the core foundation for the broad utility of Cas12b as a powerful molecular diagnostic tool [36].

Despite the universal advantages of Cas12b summarized above, existing studies still have inconsistent experimental parameters for evaluating its specificity and trans-cleavage efficiency. Different laboratories adopt disparate reaction temperatures, ion concentrations and reporter probe lengths, leading to incomparable detection limit data across publications. In addition, few studies systematically compare the signal-to-noise ratio differences between Cas12b, Cas9 and Cas12a under identical testing conditions, which makes it difficult to objectively quantify the unique superiority of Cas12b in actual clinical detection scenarios.

### 2.4. Comparison with Other Cas Variants

From structural and functional perspectives, Cas12b surpasses other Cas systems in several key aspects. Its compact molecular weight enhances cellular delivery and operational flexibility while maintaining stability under diverse thermal conditions [12]. Furthermore, Cas12b is capable of high target specificity, though its off-target propensity is dependent on guide RNA design and experimental conditions, representing a critical advantage for precise gene-editing and accurate diagnostic detection [37]. These attributes position Cas12b as a versatile tool for applications requiring robustness and reliability. Table 2 summarizes the structural differences, functional characteristics and practical operation gaps between Cas12b and other representative Cas proteins. Most existing comparative studies only evaluate Cas proteins under pure buffer environments without introducing complex clinical matrix interference, and the performance gaps between Cas12a, Cas9 and Cas12b derived from different literature cannot be cross-compared due to inconsistent experimental parameters such as temperature and spacer length, which limits the objective reference value of existing horizontal comparison data.

## 3. Applications of CRISPR-Cas12b in Pathogen Detection

CRISPR-Cas12b technology has emerged as a powerful tool for detecting various pathogens, including viruses, bacteria, and parasites. Its ability to provide rapid, sensitive, and specific detection makes it particularly valuable in clinical diagnostics and public health. The versatility of the CRISPR-Cas12b system allows for the development of diagnostic assays that can be tailored to detect a wide range of pathogens, thereby enhancing disease surveillance and management efforts. The key applications are systematically summarized in Table 3. On this basis, the design logic, performance breakthroughs, and translational value of this technology in different application scenarios are deeply analyzed.

### 3.1. Virus Diagnosis: From Single Detection to Dynamic Monitoring of Virus Variants

In the field of virus diagnosis, the CRISPR-Cas12b system demonstrates unique application potential. The high thermal stability of this system enables seamless integration with isothermal amplification technology, significantly enhancing detection efficiency. For instance, in SARS-CoV-2 detection, optimization of sgRNA design has enabled accurate identification of viral variants [43]. Notably, the system’s ability to recognize single-nucleotide polymorphisms (SNPs) provides a powerful tool for tracking virus evolution [44]. Nevertheless, traditional nucleic acid amplification-dependent detection techniques have inherent drawbacks, including aerosol contamination risks and interference from complex matrix substances. These issues are inherent to almost all detection strategies that require independent pre-amplification and separate detection operations. Additionally, the amplification-free graphene field-effect transistor (gFET) biosensor shows the potential for direct detection without relying on pre-amplification, offering a new direction for simplifying procedures and improving robustness [45]. More importantly, emerging one-pot detection strategies, which complete nucleic acid amplification and signal detection in a single closed-tube system, can effectively avoid the cross-contamination and matrix interference associated with open-tube, separate operations [44]. Future research will focus on developing and optimizing two promising CRISPR detection strategies: (1) novel one-pot CRISPR detection systems that integrate amplification and detection in a closed-tube reaction to avoid cross-contamination and matrix interference, and (2) amplification-free CRISPR detection strategies that completely eliminate the reliance on pre-amplification, thereby fundamentally circumventing the inherent limitations of traditional pre-amplification-dependent detection methods.

However, current research still has certain limitations. Most detection systems are designed for single viruses, making it difficult to cope with common mixed-infection scenarios in clinical practice. This limitation mainly stems from the complexity of orthogonal sgRNA design. To address this bottleneck, researchers have started to explore a new strategy based on PAM sequence engineering. By designing Cas12b variants that specifically recognize different PAMs, researchers have provided a new solution for multiplex detection [46,47]. Existing virus detection studies based on CRISPR-Cas12b exhibit considerable inconsistencies in experimental standards. Different teams adopt disparate sgRNA lengths, amplification primer concentrations, and one-pot reaction temperatures, leading to inconsistent reported sensitivities for multiplex detection. Furthermore, most multiplex virus assays have only been verified using artificial mixed nucleic acid samples, and large-scale clinical validation using mixed-infection specimens is lacking to support their practical clinical application.

### 3.2. Bacterial Diagnosis: Technological Innovations Driving Clinical Applications

Notable progress has been made in bacterial diagnosis using CRISPR-Cas12b technology. Traditional culture methods require a detection cycle of several days, while the Cas12b-based detection method can shorten this process to a few hours. In the detection of food-borne pathogens, the interference of complex food matrices has been effectively addressed by introducing the Uracil-DNA Glycosylase (UDG) anti-contamination system and immunomagnetic bead enrichment technology, enabling highly sensitive detection of pathogens such as *Salmonella* [48,49]. There are significant deficiencies in the cost–benefit assessment of existing anti-interference strategies. Research indicates that the introduction of UDG enzyme significantly increases the cost per reaction, while the immunomagnetic bead enrichment operation requires approximately 30 min of additional detection time [49]. However, current research mostly focuses on improving technical performance, neglecting the balance between cost and efficiency in different application scenarios.

Regarding the diagnosis of respiratory pathogens, this technology demonstrates unique advantages. By optimizing sample processing methods and improving nucleic acid extraction, researchers have achieved stable detection even in sputum samples with low pathogen loads [50]. Of particular note is the system’s ability to detect free microbial DNA in plasma [51], which has opened new avenues for the diagnosis of extrapulmonary infections. Most published bacterial detection schemes adopt self-matched sample pre-processing kits and customized nucleic acid extraction reagents, so their detection limits cannot be uniformly compared across different studies. In addition, few studies carry out comprehensive cost and throughput evaluation oriented to grassroots clinical and food supervision platforms, which makes it difficult to judge the practical promotion value of each anti-interference strategy under actual industrial and medical conditions.

### 3.3. Parasite Diagnosis: Breaking Through the Limitations of Traditional Detection Methods

Parasite diagnosis has long faced unique technical challenges stemming from complex parasitic genomes and severe matrix interference in clinical and environmental samples. Unlike viral and bacterial detection, parasitic assays are particularly susceptible to interference due to multiple inherent factors: clinical samples such as feces and tissues contain abundant inhibitory substances and substantial host background DNA, while parasite target DNA usually accounts for an extremely low proportion of total genomic DNA. In addition, parasite genomes contain large numbers of repetitive sequences and homologous genetic regions, and frequent clinical mixed infections further increase the difficulty of specific detection, making anti-interference capability a core and unique requirement for parasitic diagnostic techniques. CRISPR-Cas12b technology demonstrates prominent advantages in addressing these challenges. Different from conventional PCR, which relies primarily on primer annealing for preliminary amplification screening, the CRISPR-Cas system achieves secondary sequence verification through high-precision sgRNA base pairing. Benefiting from the stringent mismatch sensitivity of the seed region, Cas12b can effectively distinguish single-base differences in complex homologous sequences, exhibiting stronger matrix interference tolerance and higher detection specificity than traditional PCR even under pre-amplification-dependent conditions. In the detection of *T. gondii*, the system’s excellent tolerance to complex environmental matrices such as soil and feces enables reliable parasitic detection in complex backgrounds and renders it an ideal tool for environmental monitoring [52]. In the detection of *Trypanosoma brucei*, its attomolar-level ultrahigh sensitivity supports accurate identification of trace parasitic nucleic acids, enabling early-stage infection diagnosis [53].

Nevertheless, the highly repetitive and complex characteristics of parasite genomes still pose special obstacles to high-specificity sgRNA design. To address this problem, researchers have proposed optimized target design strategies tailored to parasitic genomic features. Preferential selection of single-copy specific genes and highly species-conserved genomic regions effectively avoids non-specific binding caused by homologous sequences, substantially improving detection specificity for complex parasitic templates. Selecting single-copy targets reduces off-target risk originating from multiple homologous genomic loci [54,55].

Although most current CRISPR-Cas12b detection workflows still rely on pre-amplification to compensate for the limitation of intrinsic single-step detection sensitivity, such amplification procedures only serve to enrich target copy numbers. The core specific recognition and anti-interference capability still depends on the secondary screening of Cas12b–sgRNA complexes, which fundamentally differentiates it from conventional PCR relying solely on primer design for conserved regions. Furthermore, to circumvent the inherent contamination and matrix interference drawbacks of traditional pre-amplification-dependent detection, future research will focus on developing and optimizing advanced CRISPR diagnostic strategies, including high-efficiency one-pot closed-tube detection systems and amplification-free CRISPR sensing techniques, to further improve the practicability and robustness of parasitic detection. These innovative methods provide solid technical support for the establishment of a comprehensive prevention and control framework for parasitic diseases. Existing studies on Cas12b-based parasite detection lack unified evaluation standards for anti-interference performance. Different research groups adopt different extraction kits, amplification parameters, and sgRNA design schemes, leading to incomparable sensitivity and specificity data across research. Moreover, most relevant experiments test only single parasite species, and few studies have systematically explored simultaneous detection of multiple parasites in co-infected clinical specimens.

## 4. Discussion

Collectively, the application cases outlined above demonstrate that the CRISPR-Cas12b system offers distinct technical advantages and promising translational potential in molecular diagnostics. However, this technology is still at the stage of laboratory exploration and methodological verification. To truly transform this technology into a stable, universal, and implementable clinical diagnostic tool, several technical shortcomings and application limitations must still be overcome. Identifying and summarizing the core issues based on existing research findings, and proposing targeted optimization strategies, will guide the mature implementation of Cas12b-based detection technology. Nevertheless, many underlying scientific questions remain unresolved. For example, how sequence context modulates Cas12b cis- and trans-cleavage coupling is still not fully understood; clarifying these molecular mechanisms will lay a theoretical foundation—rather than relying solely on empirical optimization—for next-generation assay design.

In actual detection scenarios, the low recognition efficiency of low-abundance targets in complex samples is currently the primary issue restricting the improvement of Cas12b detection performance. Clinical samples have complex matrices containing numerous endogenous interfering substances. In addition, pathogen loads vary among individuals, which can affect the stability and reliability of the detection system. Therefore, it is essential to carry out fine-grained optimization of the detection system. From the perspective of sgRNA design, considering the molecular characteristics of Cas12b, a length of 18–20 nucleotides is the optimal design range [16,56]. In practice, it is necessary to first identify the TTN-type PAM sequence specific to Cas12b, and preferentially select highly conserved regions of the pathogen genome to design the targeting sequence, so as to ensure detection specificity and minimize the off-target risk. Current sgRNA optimization mostly relies on trial-and-error screening. A major unsolved bottleneck is the lack of predictive models that can quantitatively evaluate how single-base mismatches and flanking sequences affect discrimination performance. Future work should prioritize building guide-prediction tools tailored for Cas12b biochemical properties, instead of directly transplanting frameworks from other well-characterized Cas effectors.

The current mainstream CRISPR design platforms, including CRISPR-DT and CasFinder, are difficult to fully adapt to the unique biochemical characteristics of Cas12b. Most of these tools are developed based on the mechanisms of action of Cas9 and Cas12a, without fully accounting for Cas12b’s unique 5′-TTN PAM preference and the distinct structural constraints imposed by its sgRNA scaffold. As a result, the sgRNAs designed in batches often have problems such as insufficient activity and poor targeting efficiency, and a large number of experiments are required for screening and verification, which invisibly increases the experimental cycle and research and development costs. Notably, Wang et al. [56] demonstrated that the split-sgRNA strategy for modular engineering of long sgRNAs enables precise modulation of Cas12b activity while preserving its trans-cleavage activity. This strategy has been successfully applied to the direct detection of microRNA. This progress marks a shift in the research focus from the development of general-purpose tools to specialized solutions tailored to the characteristics of Cas12b. Even with split-sgRNA engineering, the quantitative relationship between guide modular structure and collateral activation is not completely understood. This represents an important open scientific question. Future priority directions include combining structural biology and machine learning to realize rational, high-throughput sgRNA design exclusive for Cas12b diagnostics.

In the nucleic acid amplification step, an appropriate technique needs to be selected according to the sample type. For samples with complex components, LAMP has stronger tolerance and is more suitable. In on-site detection where speed and portability are pursued, recombinase polymerase amplification (RPA) is more applicable. However, the combination of LAMP and RPA with Cas12b still requires optimization. The high-concentration primers of LAMP may bind non-specifically to Cas12b, suppressing its trans-cleavage activity [28]. The optimal reaction temperatures of RPA and Cas12b are mismatched (37–42 °C for RPA, 45–55 °C for Cas12b). Moreover, premature cis-cleavage activity of Cas12b will degrade RPA amplicons and severely suppress amplification efficiency, while some components in the RPA mixture can inhibit Cas12b function. To overcome these incompatibilities, most existing studies adopt separate stepwise reactions of amplification followed by detection, which contradicts the original design concept of convenient one-tube one-pot testing [30,56]. The mechanistic origins of mutual inhibition between isothermal amplification reagents and Cas12b nuclease remain incompletely characterized. Breaking this incompatibility barrier is a key technical bottleneck for true one-pot POCT. Future research should focus on buffer reformation, enzyme engineering, and spatiotemporal reaction control to achieve seamless integration of amplification and Cas12b cleavage in single-tube systems.

The insufficient multiplex detection ability and the lack of a standardized system pose another obstacle. Existing methods are mostly limited to single-target detection and are difficult to meet the practical needs such as mixed infections. Although some studies have attempted to achieve multiplex detection by combining different Cas proteins, there are still bottlenecks in aspects such as orthogonal sgRNA design and signal channel discrimination [57]. More crucially, there is currently a lack of a “standardized operating procedure for CRISPR-Cas12b detection”. Sample processing, reaction system formulation (such as the concentration ratio of Cas12b to sgRNA), and result interpretation criteria (such as fluorescence threshold setting) are all defined by individual laboratories. This has led to significant differences in the detection results of the same pathogen across different studies. For instance, the reported detection limits of *Mycobacterium tuberculosis* vary from 0.8 fg/μL to 5 fg/μL in different research, making it impossible to establish a unified technical evaluation standard [51,58]. In the future, efforts should be made to develop a platform for the simultaneous detection of multiple pathogens. For example, by constructing a library of Cas12b PAM recognition sequence variants and integrating it with microfluidic chip technology, “single-tube multiplex detection” can be achieved [47]. At the same time, establishing unified technical standards and performance verification specifications and clarifying key quality control parameters are important foundations for promoting technology transfer. Determining how to realize true orthogonality among multiple Cas12b variants without signal crosstalk is still an unsolved scientific challenge. Besides multiplex-assay development, formulating consensus benchmark standards is an urgent practical priority; without unified reference materials and evaluation metrics, cross-laboratory performance comparison and clinical translation will be severely hindered.

The third challenge concerns the feasibility of on-site applications, with the core being the balance among cost, stability, and operational convenience. Currently, the preparation costs of Cas12b protein and sgRNA are relatively high, the stability of freeze-dried preparations at room temperature is insufficient, and the numerous operation steps limit the promotion of this technology in resource-limited scenarios. Countermeasures include developing a low-cost protein expression system, optimizing the formulation process to improve stability, and using microfluidic technology to integrate and automate the detection process. The portable detection device developed by taking advantage of the relatively small molecular weight of Cas12b provides the possibility of achieving the “sample-in-result-out” on-site detection mode [59]. The molecular rules governing Cas12b thermostability and lyophilization tolerance are not fully resolved at present. Future priorities cover directed evolution for higher stability, low-cost large-scale enzyme production, and fully integrated microfluidic systems that can simplify clinical sample pretreatment for real-world POCT scenarios.

The development path refined from practice as described above provides specific ideas for breaking through the current technical bottlenecks and also points the way for the CRISPR-Cas12b system to move from the laboratory to clinical applications. Through interdisciplinary collaboration and technology integration, this system is expected to play a more important role in areas such as infectious disease prevention and control, food safety, and environmental monitoring.

## 5. Conclusions and Future Prospects

CRISPR-Cas12b manifests unique merits for pathogen nucleic acid detection, including favorable thermal adaptability and single-base resolution. It acts as a complementary molecular diagnostic tool rather than a simple substitute for Cas9 and Cas12a, expanding available technical options for grassroots point-of-care testing, food-safety screening and environmental monitoring. Multiple proof-of-concept studies have verified its capacity for identifying viral, bacterial and parasitic targets under complex-matrix conditions.

Nevertheless, substantial scientific puzzles and technical bottlenecks still hinder its large-scale clinical deployment. At present, multiplex detection schemes are mostly adapted from other CRISPR systems, lacking Cas12b-oriented optimization. Standardized operating protocols, dedicated quality-control reference materials, and unified performance evaluation benchmarks remain absent, resulting in poor inter-laboratory reproducibility. Moreover, high enzyme production cost and insufficient batch-to-batch stability further restrict real-world popularization.

Looking forward, future research should advance along three priority dimensions: fundamental mechanism exploration, technical system innovation, and standard-specification construction. It is necessary to first further dissect the molecular mechanism of Cas12b cis-trans cleavage coupling and variant-dependent biochemical behaviors, providing theoretical support for rational assay design; second, develop temperature-regulated multiplex detection strategies, tailor-made sgRNA prediction algorithms, and integrated microfluidic portable devices to realize simplified “sample-in-result-out” workflows; third, promote the establishment of exclusive standard specifications covering reaction conditions, quality-control indexes and result-judging criteria for Cas12b-based diagnostics. With continuous breakthroughs in the above-mentioned directions, Cas12b will become a valuable technical option for public-health prevention, precision diagnosis and environmental monitoring.

## Figures and Tables

**Table 2:** Comparative Analysis of Structural, Functional, and Operational Characteristics Across CRISPR-Cas Systems.

Category	Cas9	Cas12a	Cas12b	Cas13	Cas14
Type	II	V	V	VI	V
Protein Molecular Weight	~160 kDa (e.g., SpCas9)	~130–140 kDa (e.g., AsCas12a, LbCas12a)	~110–130 kDa (e.g., AapCas12b, BvCas12b)	~130–150 kDa (e.g., LwCas13a, PspCas13b)	~64.9 kDa (e.g., Cas14a)
RNA Dependence	crRNA + tracrRNA	crRNA	crRNA + tracrRNA	crRNA	crRNA
Target Type	dsDNA	dsDNA	dsDNA	RNA	ssDNA
PAM/PFS Sequence	3′-NGG (SpCas9)	5′-TTTV (T-rich)	5′-TTN (shorter, flexible)	Non-specific 3′ PFS	No PAM
Cleavage Mechanism	Blunt-ended dsDNA cleavage (HNH + RuvC domains)	Staggered dsDNA cleavage (RuvC domain)	Staggered dsDNA cleavage (RuvC domain)	ssRNA cleavage (HEPN domain)	ssDNA cleavage (RuvC domain)
Trans-cleavage activity	None	Yes (on ssDNA)	Yes (on ssDNA)	Yes (on ssDNA)	None
Key Advantages	Mature technology and wide applicability High editing	No tracrRNA needed Enables multiplexed detection	High thermal stability High specificity Compact size	High sensitivity for RNA No reverse transcription needed for RNA viruses	Ultra-small size Specific for ssDNA targets
Key Limitations	High off-target risk Large size hinders delivery Strict PAM requirement	Lower cleavage efficiency Thermolabile T-rich PAM limit targets	Requires temperature optimization Less mature for multiplexing Specific PAM requirement	Restricted to RNA targets High false-positive risk from robust collateral activity RNase-sensitive	Limited to ssDNA targets No collateral activity limits signal amplification Less commercially available
Reference	[38]	[39]	[27,40]	[41]	[42]

Note: crRNA: Clustered Regularly Interspaced Short Palindromic Repeats RNA; tracrRNA: Trans-activating CRISPR RNA; dsDNA: Double-Stranded DNA; ssDNA: Single-Stranded DNA; PAM: Protospacer Adjacent Motif; HNH: HNH endonuclease domain; RuvC: RuvC-like endonuclease domain; HEPN: Higher Eukaryotes and Prokaryotes Nucleotide-binding domain.

**Table 3:** Summary of Main Application Examples of the CRISPR-Cas12b System.

Pathogen	Assay Mode	Detection Limit	Detection Time	Note
SARS-CoV-2	Fluorescence	5 copies/μL	25–45 min	Photo-controlled one-pot RT-LAMP-Cas12b assay targeting the N gene, without cross-reactivity to other coronaviruses, with clinical sensitivity of 97.0% and specificity of 100%.
Monkeypox virus (MPXV)	Fluorescence	4 copies/reaction	45 min	Single-tube two-step MIRA-Cas12b assay targeting the F3L gene, avoiding aerosol contamination caused by tube opening, with no cross-reactivity to other orthopoxviruses.
Canine parvovirus (CPV)	Fluorescence	10 copies/μL	45 min	A single-tube LAMP-Cas12b assay targeting the conserved VP2 gene, free of cross-reaction with other canine pathogens for point-of-care veterinary detection.
Hepatitis B virus (HBV)	Fluorescence	25 copies/mL	60 min	One-pot LAMP-Cas12b assay targeting conserved HBV DNA region, with no cross-reactivity to other blood-borne pathogens and favorable clinical performance.
African swine fever virus (ASFV)	Fluorescence, Lateral flow	8 copies/μL	60 min	Multiplex orthogonal RPA-Cas12b/Cas13a system targeting B646L and MGF505-2R genes, capable of distinguishing wild-type and gene-deleted ASFV strains.
*Salmonella*	Fluorescence	12.5 copies/reaction	60 min	One-tube LAMP-Cas12b assay targeting conserved gene, with 100% inclusivity and exclusivity for strains, suitable for food safety surveillance.
*Vibrio parahaemolyticus*	Fluorescence	1 × 10^2^ CFU/mL	25–45 min	One-pot UDG-LAMP-Cas12b system preventing carry-over contamination, consistent with qPCR results for detection of seafood samples.
*Campylobacter jejuni*	Fluorescence	10 CFU/g	40 min	An amplification-free Cas12b assay targeting species-specific genomic signatures, with higher sensitivity than traditional bacterial isolation methods.
*Mycobacterium tuberculosis*	Fluorescence	0.8 copies/μL	60 min	One-pot cross-priming amplification-Cas12b assay targeting IS6110 gene, applicable to direct detection of MTB in sputum specimens.
*Bordetella pertussis*	Fluorescence	0.2 copies/μL	30 min	One-pot RAA-Cas12b assay enabling extraction-free nucleic acid detection, avoiding aerosol contamination and exhibiting reliable clinical diagnostic performance.
*Pseudomonas aeruginosa*	Fluorescence, Lateral flow	50 CFU/reaction	60 min	Single-tube two-step RPA-Cas12b assay targeting lasR gene, applicable to rapid screening of water and environmental samples.
*Klebsiella pneumoniae*	Fluorescence	50 copies/reaction	40 min	A one-pot one-step CRISPR-AapCas12b platform targeting species-specific conserved sequence, with high specificity for identification of Klebsiella pneumoniae.
*Fusarium oxysporum*	Fluorescence	88.9 copies/reaction	45 min	One-pot LAMP-Cas12b assay targeting conserved gene, used for rapid diagnosis of soil-borne fusarium wilt in plants.
*Colletotrichum siamense*	Fluorescence	10 copies/reaction	30 min	One-pot LAMP-Cas12b assay targeting species-specific conserved gene for early diagnosis of strawberry anthracnose.
*Toxoplasma gondii*	Fluorescence, Lateral flow	10 copies/μL	30 min	Single-tube LAMP-Cas12b assay targeting B1 gene, covering multiple genotypes for zoonotic pathogen environmental monitoring.
*Trypanosoma brucei*	Fluorescence	20 pg gDNA/reaction	60 min	One-pot RPA-Cas12b assay targeting RoTat 1.2 VSG gene, suitable for point-of-care veterinary diagnosis of trypanosome infection.

## Data Availability

No new data were created or analyzed in this study. Data sharing is not applicable to this article.
